# Preventing peri-implantitis with Ag-Est816 nanocomposite: modified rat model and preventive assessment

**DOI:** 10.3389/froh.2026.1781671

**Published:** 2026-03-31

**Authors:** Zelda Ziyi Zhao, Mengyin Luan, Chun Hung Chu, Jing Zhang

**Affiliations:** 1 Faculty of Dentistry, The University of Hong Kong, Hong Kong, Hong Kong SAR, China; 2 Stomatological Hospital and College, Key Laboratory of Oral Diseases Research of Anhui Province, Anhui Medical University, Hefei, Anhui, China

**Keywords:** biofilms, *N*-acyl-homoserine lactones, oral microbiota, peri-implantitis, quorum sensing, silver nanoparticles

## Abstract

**Background:**

Peri-implantitis is a prevalent complication leading to progressive bone loss around dental implants, with current therapeutic strategies offering limited preventive efficacy.

**Objective:**

This study aimed to develop a modified rat model of peri-implantitis to evaluate the effects of a silver nanoparticle and quorum quenching enzyme nanocomposite (Ag-Est816) on peri-implant bone health.

**Methods:**

The research comprised two parts: (A) development of the peri-implantitis rat model, and (B) assessment of Ag-Est816's preventive effect. In Part A, titanium implants infected with *Porphyromonas gingivalis* and *Streptococcus sanguinis* were surgically placed into the femurs of five male Sprague-Dawley rats weighing 180–220 g. After four weeks, peri-implant bone loss was evaluated using micro-computed tomography (Micro-CT) to measure bone volume to total volume ratio (BV/TV), and histological analysis was performed to assess bone architecture. Non-infected implants served as controls. In Part B, Ag-Est816 was applied to infected titanium implants, while control implants were treated with sterilized phosphate-buffered saline (PBS). Micro-CT and histological analysis were used to evaluate bone loss and tissue integrity.

**Results:**

In Part A, the BV/TV of infected implants and controls was 6.9 ± 1.4% vs. 32.8 ± 9.0% (*p* < 0.001). Histological analysis revealed disorganized collagen, impaired mineralization, and stalled bone formation at the implant interface in the infected samples, in contrast to the ordered, mature osseointegration observed in the controls. In Part B, the BV/TV of Ag-Est816-treated implants and controls was 23.6 ± 3.3% vs. 5.1 ± 1.8% (*p* < 0.001). Histological analysis revealed new bone regeneration with trabecular integrity around Ag-Est816-treated implants, in contrast to compromised osseointegration with disrupted, sparse bone and fibrous, inflammatory tissue in the controls.

**Conclusions:**

A modified rat femur model of peri-implantitis was successfully developed. Ag-Est816 effectively prevented peri-implant bone loss.

## 1 Introduction

Dental implants have revolutionized modern dentistry, offering predictable solutions for tooth loss and restoring oral function. However, the long-term success of implants is threatened by peri-implantitis, a biofilm-driven inflammatory condition characterized by soft tissue inflammation and progressive bone loss around the implant. With a prevalence of approximately 20% at patient-level and 11.5% at implant-level, peri-implantitis poses a major clinical and economic challenge (1). Left untreated, it leads to implant failure, necessitating complex regenerative surgeries or explantation. The pathogenesis of peri-implantitis is closely linked to dysbiotic microbial communities. While “red complex” pathogens such as *Porphyromonas gingivalis* (*P. gingivalis*) are considered keystone species, the biofilm initiation facilitated by early colonizers like *Streptococcus sanguinis* (*S. sanguinis*) is a critical prerequisite for subsequent dysbiosis and disease progression (2–7). These structured biofilms evade host immune responses and resist conventional therapies, perpetuating inflammation and osteoclast-mediated bone resorption (8).

Current preventive and therapeutic approaches for peri-implantitis focus on mechanical debridement, antiseptic rinses, and systemic or local antibiotics (9). While mechanical cleaning disrupts biofilms temporarily, it often fails to eliminate subgingival pathogens or prevent rapid biofilm reformation (10). Antimicrobial agents like chlorhexidine and hydrogen peroxide exhibit limited efficacy due to poor penetration into biofilms and the risk of microbial resistance. Systemic antibiotics, though effective in acute phases, are unsuitable for long-term prevention and contribute to the global crisis of antibiotic resistance (11). Furthermore, these strategies do not address the quorum sensing (QS) mechanisms that regulate biofilm formation, virulence factor production, and bacterial communication-a critical oversight in managing peri-implant infections (12).

Recent advances in nanotechnology and anti-virulence therapies offer novel avenues to combat peri-implantitis (13, 14). Silver nanoparticles (AgNPs) have gained attention for their broad-spectrum antimicrobial properties, disrupting bacterial membranes and generating reactive oxygen species (15). However, standalone AgNPs lack specificity and may provoke cytotoxicity at high concentrations (16). To enhance precision, researchers are exploring combinatorial approaches that pair AgNPs with enzymes capable of quorum quenching-the disruption of bacterial QS signaling. Quorum quenching enzymes, such as acyl-homoserine lactone lactonases (e.g., Est816), degrade signaling molecules like autoinducers-1, thereby inhibiting biofilm formation and virulence without exerting selective pressure for resistance (17, 18). Integrating AgNPs with quorum quenching enzymes could synergistically target both bacterial viability and their pathogenic communication networks.

Despite promising *in vitro* studies, the translation of nanoparticle-enzyme composites to *in vivo* peri-implantitis models remains underexplored (19). Existing research has focused on either antimicrobial efficacy or QS inhibition in isolation, neglecting the potential of dual-action strategies to address biofilm resilience and host tissue damage simultaneously. Moreover, few studies have evaluated the osteoprotective effects of such nanocomposites in the context of peri-implant bone loss. The Ag-Est816 nanocomposite, which combines AgNPs with the quorum quenching enzyme Est816, represents a novel solution. The silver component targets bacterial cells directly, while Est816 disrupts QS pathways, preventing biofilm maturation and reducing inflammation-driven bone resorption. This dual mechanism could overcome the limitations of conventional therapies by providing sustained, localized antimicrobial action and modulating the pathogenic biofilm microenvironment. The aim of the study was to prevent peri-implantitis using a silver nanoparticle-based nanocomposite containing quorum quenching enzyme (Ag-Est816).

## 2 Materials and methods

### 2.1 Development and validation of peri-implantitis rat model

**Figure 1:**
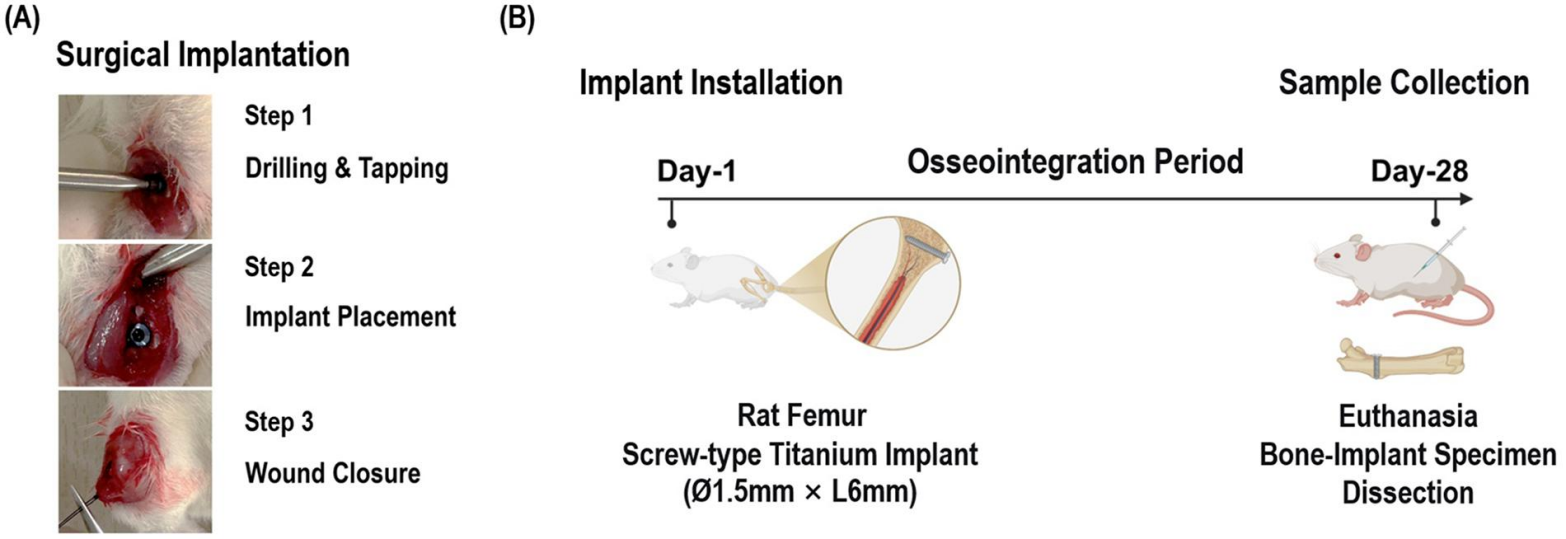
Schematic illustration of the rat femoral implant model establishment. **(A)** The surgical implantation process was showed as three steps. **(B)** The implant was left for 28 days to allow for osseointegration. The rats were then euthanized for study. Created in Bio Render. Zhao, Z. (2026) https://BioRender.com/z10v9cp.

This animal study was performed in mainland China. Sprague-Dawley rats were purchased from Animal Experimental Center of Anhui Medical University, Anhui, China. Peri-implantitis was induced by implanting titanium rods (1.5 mm diameter and 6 mm length) into the rats’ femurs (one rod on each side) for four weeks. Figure 1 shows a schematic illustration of the rat femoral implant model establishment. The study protocol was conformed to the ARRIVE guidelines of the Use of Laboratory Animals of the National Institutes of Health (20). The study was approved by the animal ethics committee of Anhui Medical University, China (Protocol No. LLSC20242476).

Ten male Sprague-Dawley rats weighing 180–220 g were randomly allocated to the following two groups:

1. Non-infected group: implant with no bacterial contamination;
2. Infected group: implant contaminated with *P. gingivalis* and *S. sanguinis*.

Implant was surgically placed in three steps: Step 1, drilling and tapping of the femur; Step 2, placement of the titanium implant; Step 3, wound closure. The implant was left for 28 days to allow for osseointegration. Rats were euthanized by anesthetic overdose at the end of the experimental period. Anesthetic overdose was induced by intraperitoneal injection of a mixture of xylazine hydrochloride (10 mg kg^−1^) and ketamine hydrochloride (40 mg kg^−1^). The depth of anesthesia, confirmed by the loss of pedal and corneal reflexes, was maintained. Subsequently, to ensure euthanasia, a lethal overdose of the same anesthetic mixture (xylazine hydrochloride: 30–50 mg kg^−1^, ketamine hydrochloride: 120–200 mg kg^−1^) was administered intraperitoneally, resulting in cardiac arrest. Death was verified by cessation of breathing and heartbeat. Femurs with implants were then carefully harvested for further analysis.

*P. gingivalis* (ATCC 33277) was cultured anaerobically in brain heart infusion (BHI) broth (3.6% BHI broth, 0.5% yeast extract, 5.0 μg mL^−1^ hemin, and 1.0 μg mL^−1^ vitamin K1). *S. sanguinis* (ATCC 10556) was cultured anaerobically in BHI broth (3.6% BHI broth, 0.5% yeast extract). Both cultures were grown to mid-log phase and adjusted to a concentration of 1 × 10^7^ colony-forming units (CFU) mL^−1^ used in the following experiments. For implant contamination, sterile titanium rods were immersed in 1 mL of the dual-species bacterial suspension (1:1 mixture of *P. gingivalis* and *S. sanguinis*) and incubated anaerobically at 37 °C for 24 h to allow biofilm formation on the implant surface. The sterile titanium rods in the non-infected group had no treatment.

In micro-computed tomography (Micro-CT) analysis, the specimens of the femurs with titanium rods were fixed with 10% buffered formalin for 24 h and stored in 70% ethanol until scanned by a micro-CT (SkyScan 1172, Bruker Corporation, Billerica, USA). The Specimens were scanned with the following parameters: isotropic voxel size of 50 μm, x-ray source voltage of 50 kV, current of 200 *μ*A, exposure time of 500 ms, and rotation step of 0.4° over 360°. Images were reconstructed and the three-dimensional morphometric analysis was conducted using CT Analyzer software (SkyScan CTAn, Bruker Corporation, Billerica, USA). The bone volume/tissue volume (BV/TV, %) and trabecular number (Tb. N, mm^−1^) were quantified. Two examiners previously trained and calibrated, independently measured and averaged the results. Repeated measurement and personnel comparison of the measured samples showed no significant difference. The examiners were blinded to the group allocation.

After Micro-CT analysis, the samples were washed under running water and dehydrated using ascending grades of alcohol and xylene, infiltrated, and embedded in methyl methacrylate for non-decalcified sectioning. The central section was ground to a final thickness of 50 μm and treated with methylene blue-acid fuchsin staining and Masson's trichrome staining respectively. Image acquisition was performed with an optic microscope (DMi8, Leica Camera AG, Wetzlar, Germany). Histological evaluation was conducted independently by two experienced examiners blinded to the experimental groups.

### 2.2 Application of Ag-Est816 nanocomposite

After developing the peri-implantitis rat-model, the model was used to evaluate the effect of Ag-Est816 on peri-implantitis. The Ag-Est816 was synthesized under light-protected conditions via a one-pot co-reduction and coordination method. Briefly, 25.5 mg of branched polyethylenimine (BPEI, Mw 40 kDa) dissolved in 16.25 mL of deionized water was added with 8.75 mL of an Est816 solution (30 U mL^−1^), and the mixture was stirred for 30 min. Subsequently, 12.5 mL of silver nitrate solution (0.68 mg mL^−1^) was added dropwise. The reaction proceeded for 3 h at 25 °C under continuous magnetic stirring. The final nanocomposite was stored at 4 °C in a light-proof container until use. For *in vivo* application, the nanocomposite was diluted to final working concentrations of 5 µg mL^−1^ (silver) and 6 U mL^−1^ (enzymatic activity). The final working concentrations of 5 µg mL^−1^ silver and 6 U mL^−1^ enzyme activity were selected based on a comprehensive *in vitro* assessment balancing antimicrobial efficacy with host cytocompatibility, as detailed in our companion study focused on Ag-Est816 development. Specifically, this silver concentration falls within the established biocompatible range (≤ 10 µg mL^−1^) for human gingival fibroblasts (HGFs) and was confirmed to cause no significant cytotoxicity or morphological disruption to HGFs. The accompanying enzyme activity level (6 U mL^−1^) represents a dose that, when combined with 5 µg mL^−1^ Ag, achieves synergistic biofilm disruption while preserving >90% of the conjugated enzyme's quorum-quenching activity. *In vitro* antibiofilm assays against *P. gingivalis* and *S. sanguinis* demonstrated that this specific combination significantly reduced biofilm biomass, viability, and structural integrity compared to either component alone or PBS treatments. The key physicochemical parameters of the batch used are as follows: Ag-Est816 had a hydrodynamic diameter of 98 ± 5 nm (DLS) and exhibited a spherical morphology. XPS analysis confirmed the formation of N-Ag bonds and the coexistence of metallic silver (Ag⁰) and ionic silver (Ag+). The material demonstrated excellent colloidal stability across a temperature range (4–37 °C). A critical feature for sustained efficacy and safety was its controlled ion release profile, with less than 0.2% cumulative Ag+ release (ICP-MS). Sterilized phosphate-buffered saline (PBS) served as the controls. Ten rats were randomly and evenly allocated to the following two groups:

1. PBS group: *P. gingivalis* and *S. sanguinis* treated with PBS;
2. Ag-Est816 group: *P. gingivalis* and *S. sanguinis* treated with Ag-Est816.

The titanium rods were previously soaked in *P. gingivalis* and *S. sanguinis* suspension co-cultured with PBS or Ag-Est816 (concentrations of silver reached to 5 μg mL^−1^ with enzyme activity of 6 U mL^−1^) before being placed into the rats’ femurs. The process of Micro-CT analysis and histological staining were described above.

To preliminarily evaluate the potential systemic toxicity of Ag-Est816, major organs (heart, liver, spleen, lungs, and kidneys) were harvested from rats in the non-infected group and the Ag-Est816-treated group. The organs were fixed in 10% neutral buffered formalin for 48 h, processed through graded alcohols and xylene, and embedded in paraffin. Sections of 5 μm thickness were cut and stained with hematoxylin and eosin (H&E). The stained sections were examined under a light microscope (DMi8, Leica) by two experienced examiners blinded to the group allocation. The assessment focused on identifying any pathological alterations, such as inflammation, necrosis, degeneration, or abnormal cellular infiltration, compared to the normal histoarchitecture observed in the non-infected group.

### 2.3 Statistical analysis

Statistical analyses were performed using GraphPad Prism (Version 8.0, GraphPad Software Company, San Diego, USA). To ensure statistical rigor and independence of experimental units, the animal (*n* = 5 per group) was treated as the primary unit of analysis. For each rat, the measurements from the bilateral femoral implants were averaged to provide a single representative value. The Shapiro–Wilk test was used to assess normality of the data. For comparisons between two groups, Student's t-test was used for parametric data. To account for the small sample size and to verify the robustness of the parametric results, the primary outcome (BV/TV) was also analyzed using the non-parametric Mann–Whitney U test. The significance level was set at 0.05. An *a priori* sample size calculation was performed using G*Power (version 3.1.9.7; Heinrich-Heine-Universität Düsseldorf, Germany) to determine the minimum number of animals required. The calculation was based on the primary outcome variable, BV/TV, using preliminary data from a pilot study (*n* = 3 per group) and published literature on similar animal models of peri-implantitis (21). The following parameters were used: t-test for independent means, two-tailed, effect size d = 3.5 (calculated based on an expected mean difference of 18% and pooled standard deviation of 5%), *α* = 0.05, power (1-*β*) = 0.80, and an allocation ratio of 1. The analysis indicated that 5 animals per group would be sufficient to detect a large biological effect with 80% power at a significance level of 0.05. This sample size is consistent with previous studies using similar rat femoral implant models and adheres to the 3Rs principles (Replacement, Reduction, Refinement) by minimizing animal use while ensuring adequate statistical power to detect clinically meaningful differences.

## 3 Results

### 3.1 Validation of the peri-implantitis rat model

**Figure 2:**
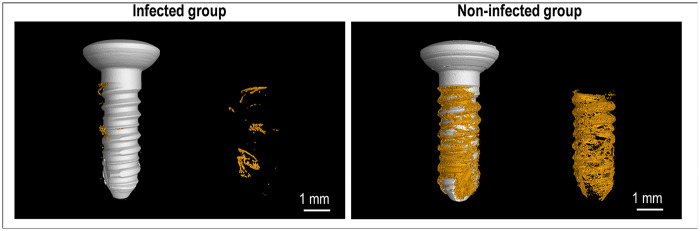
3D images of the peri-implant bone (samples were scanned with Micro-CT at a 50-μm voxel size). Left: Bone (shown in yellow) around infected implant. Right: Bone around non-infected implant.

The successful establishment of the peri-implantitis rat model was first confirmed using Micro-CT analysis. Four weeks after the implantation of bacteria-contaminated titanium rods, a significant and pronounced bone destruction was observed around the implants in infected group. Figure 2 shows representative Micro-CT scanning pictures of the peri-implant bone. Visual comparison revealed a marked reduction in bone volume, increased porosity, and disrupted trabecular architecture in infected group compared to the dense, contiguous bone observed in non-infected group.

Quantitative morphometric parameters, including BV/TV and Tb. N, were derived from these images using CT Analyzer software. Table 1 shows that the BV/TV value of infected group was significantly less than that of non-infected group (6.9 ± 1.4 vs. 32.8 ± 9.0, *p* < 0.001). Concurrently, the Tb. N value was significantly lower in infected group compared to that in non-infected group (0.6 ± 0.2 vs. 6.9 ± 1.3, *p* < 0.001). These quantitative data, coupled with the visual observation in the 3D-rendered Micro-CT images, validated the efficacy of the protocol in peri-implant bone loss.

**Table 1:** Bone volume fraction (BV/TV) and trabecular number (Tb. N) around femoral implants in the two groups (*n* = 5 rats per group; bilateral data averaged per animal).

Measurement	Infected	Non-infected	*p* value
BV/TV (%)	6.9 ± 1.4	32.8 ± 9.0	<0.001
Tb. N (mm^−1^)	0.6 ± 0.2	6.9 ± 1.3	<0.001

**Figure 3:**
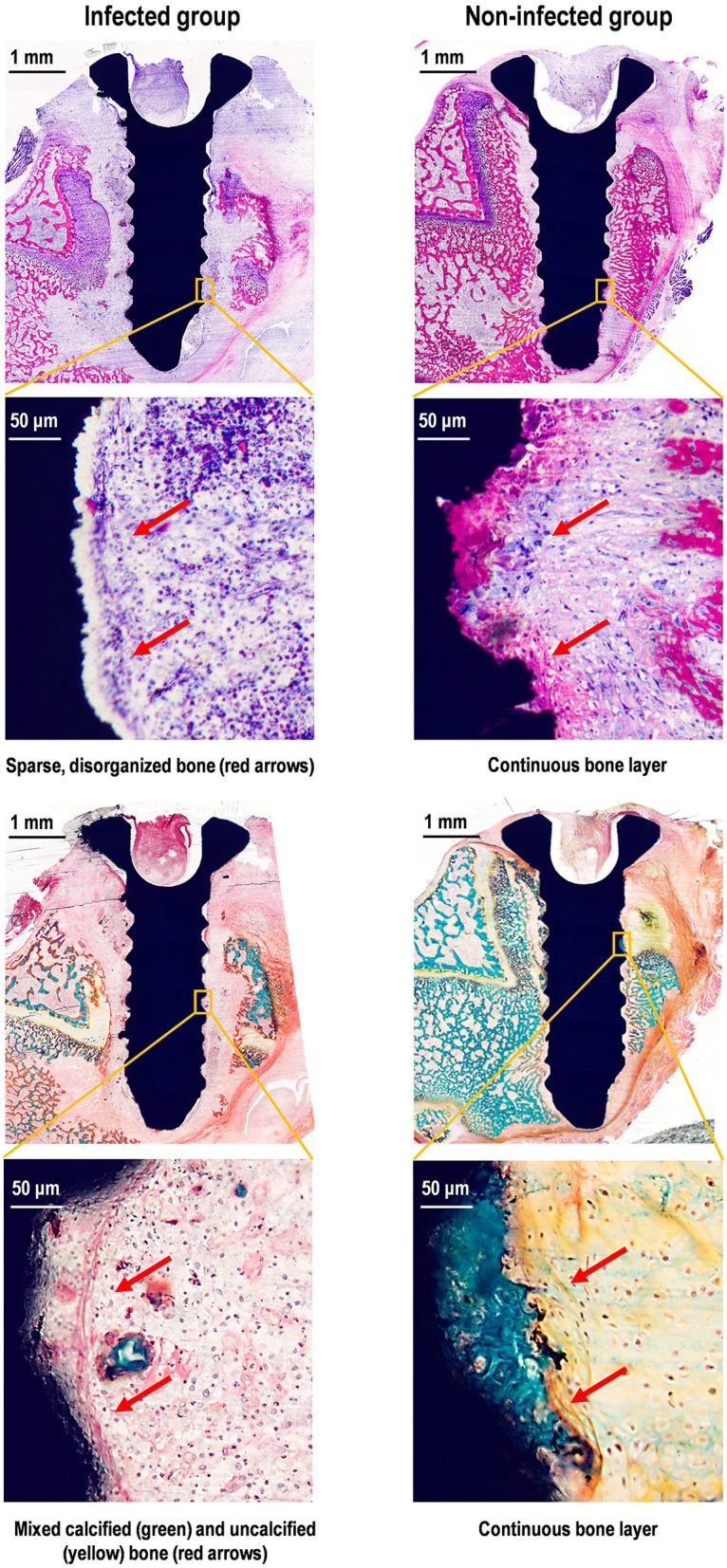
Cross sections of peri-implant bone around infected (left) and non-infected (right) implant. Upper sections: Methylene blue-acid fuchsin staining: Deep blue (osteoblast nuclei), pale purple (cytoplasm), grayish-purple (new bone). Lower sections Masson's trichrome staining: Green (mature bone), yellow/light green (immature bone).

Histological analysis using methylene blue-acid fuchsin staining and Masson's trichrome staining visually demonstrated the detrimental impact of bacterial infection on osseointegration. Figure 3 shows representative cross-sections of peri-implant bone around infected (left) and non-infected (right) implants. In infected group, methylene blue–acid fuchsin staining revealed abnormal bone healing at the implant interface, characterized by scattered dark blue osteoblast nuclei, sparse pale purple cytoplasmic matrix, and minimal grayish-purple new bone formation. The disorganized trabecular structure (red arrows) contrasted sharply with the continuous bone layer observed in non-infected group. Masson's trichrome staining further demonstrated impaired mineralization, with abundant yellow-stained uncalcified osteoid irregularly interspersed with sparse, discontinuous, green-stained calcified bone (red arrows). In contrast, non-infected group exhibited a dense, continuous zone of mineralized (green) bone surrounding the implant, indicating normal bone formation and maturation.

Overall, these results verified that pathogenic biofilms compromised successful implant integration and disrupted the bone-regenerative microenvironment.

### 3.2 Ag-Est816 reduced peri-implant bone loss

**Figure 4:**
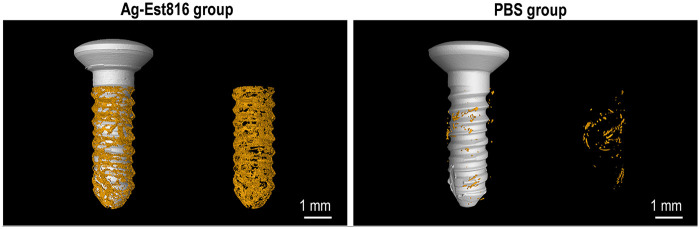
3D images of peri-implant bone with peri-implantitis (samples were scanned with Micro-CT at a 50-μm voxel size). Left: Bone (shown in yellow) around Ag-Est816-treated implant. Right: Bone around PBS-treated implant.

The protective effect of Ag-Est816 against infection-driven bone loss was comprehensively evaluated. Figure 4 shows that Ag-Est816 maintained more peri-implant bone mass.

Table 2 reveals that BV/TV value of Ag-Est816 group was significantly higher than that of PBS group (23.6 ± 3.3 vs. 5.1 ± 1.8, *p* < 0.001). Tb. N value of Ag-Est816 group was significantly higher than that of PBS group (5.7 ± 1.5 vs. 0.8 ± 0.3, *p* < 0.001).

**Table 2:** Bone volume fraction (BV/TV) and trabecular number (Tb. N) around femoral implants in the two groups (*n* = 5 rats per group; bilateral data averaged per animal).

Measurement	Ag-Est816	PBS	*p* value
BV/TV (%)	23.6 ± 3.3	5.1 ± 1.8	<0.001
Tb. N (mm^−1^)	5.7 ± 1.5	0.8 ± 0.3	<0.001

**Figure 5:**
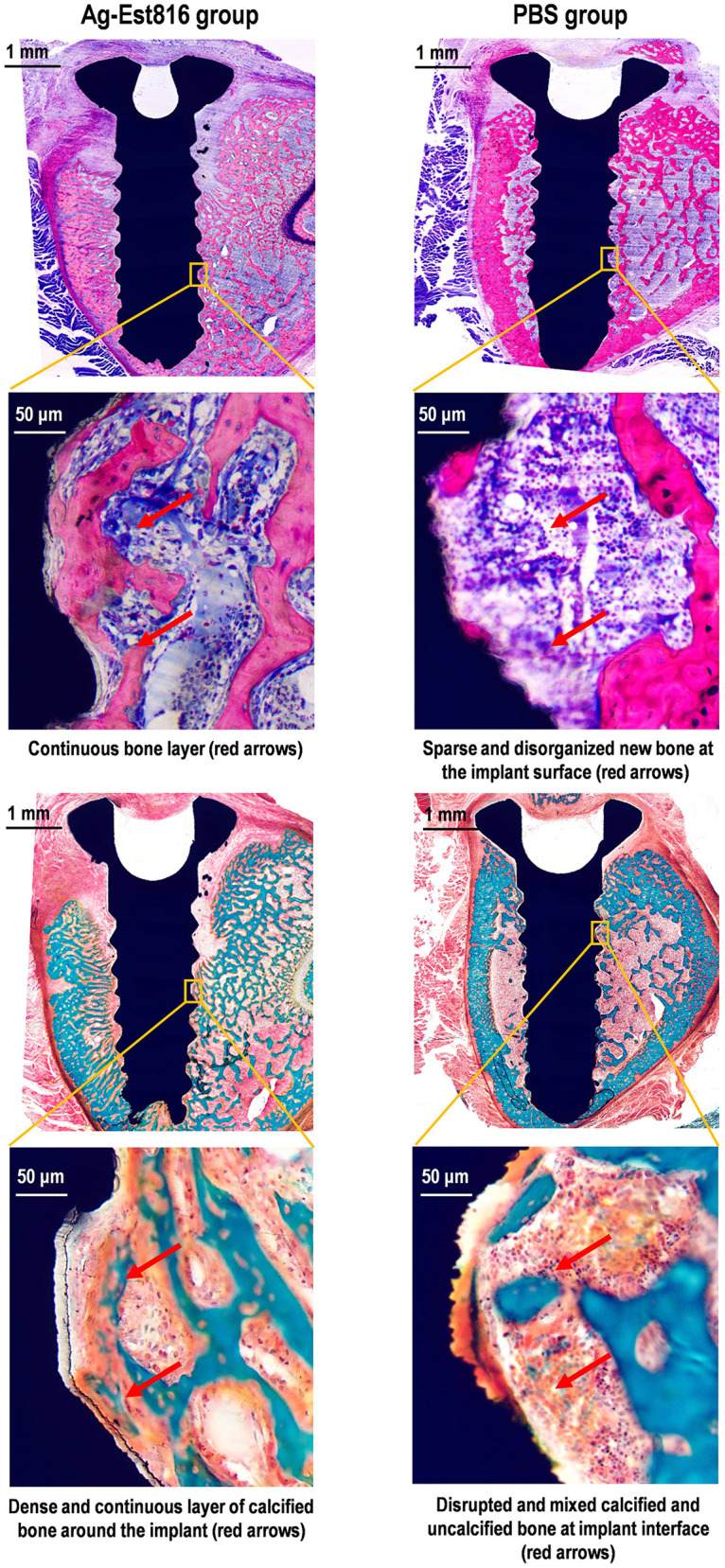
Cross sections of peri-implant bone with peri-implantitis around Ag-Est816-treated (left) and PBS-treated (right) implant. Upper sections: Methylene blue-acid fuchsin staining: Deep blue (osteoblast nuclei), pale purple (cytoplasm), grayish-purple (new bone). Lower sections Masson's trichrome staining: Green (mature bone), yellow/light green (immature bone).

Histological analysis supported the Micro-CT results. Figure 5 shows cross-sections of peri-implant bone with implantitis in Ag-Est816 group (left) and PBS group (right). In Ag-Est816 group, methylene blue-acid fuchsin staining revealed well-preserved trabecular bone structure, with dense, continuous bone matrix, smaller marrow spaces, and minimal inflammation. These features indicated active new bone formation near the implant, with high-magnification images showing tightly connected trabeculae and a clear boundary between bone and soft tissue. In contrast, PBS group showed significant bone loss and disruption, with large gaps filled by loose fibrous tissue and inflammatory cells, and only thin, fragmented bone remaining.

Masson's trichrome staining confirmed these findings. Implants in Ag-Est816 group had extensive blue-green new bone mixed with red mature bone and well-organized collagen fibers, indicating ongoing bone repair. Implants in PBS group showed only small amounts of blue-green osteoid on fragmented red bone, with a sparse and disorganized collagen network, reflecting poor bone healing.

**Figure 6:**
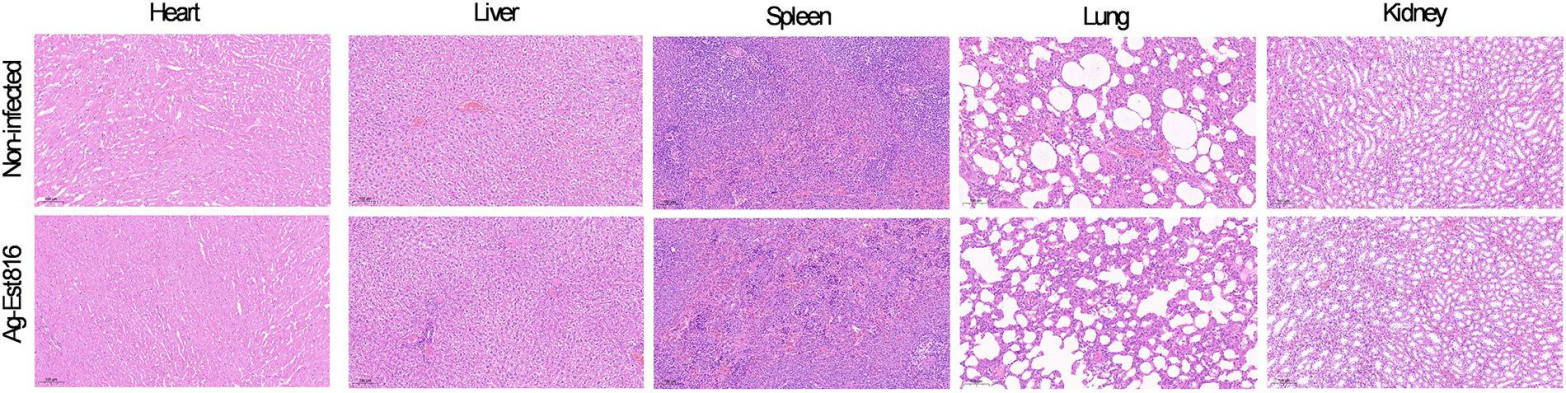
Representative H&E-stained images of major organs. Upper: non-infected group. Lower: Ag-Est816 group.

Histopathological examination of H&E-stained sections from the heart, liver, spleen, lungs, and kidneys revealed no evident signs of acute toxicity or tissue damage in rats treated with Ag-Est816. The cytoarchitecture of all examined organs from the Ag-Est816 group (Figure 6) was comparable to that of the non-infected group. Specifically, cardiac muscle fibers appeared intact with normal striations; hepatocytes exhibited regular cord-like arrangements with distinct nuclei and no signs of fatty change or necrosis; splenic white and red pulp structures were preserved; alveolar spaces in the lungs were clear without congestion or inflammatory cell accumulation; and renal glomeruli and tubules maintained normal morphology. The absence of observable pathological changes in these vital organs represented a preliminary safety assessment based on qualitative histopathological observation over a 4-week period.

Overall, these findings confirmed the protective effect of Ag-Est816 against peri-implantitis, as it preserved bone architecture and promoted new bone formation, thereby supporting successful implant integration.

## 4 Discussion

This study first established a modified rat model of peri-implantitis. This model closely mimicked early microbial colonization on titanium implants. Within this model, Ag-Est816 proved to be a highly effective agent to prevent peri-implant bone loss, thereby preserving peri-implant bone mass and structural integrity.

The establishment of a reliable animal model is a prerequisite for evaluating new strategies. Unlike previous rat models of peri-implantitis that rely on the injection of lipopolysaccharides or the use of non-oral pathogens (e.g., *Staphylococcus aureus*), this model was innovatively designed to better simulate key clinical etiologies (21, 22). First, it employed a dual-species biofilm composed of *P. gingivalis* and *S. sanguinis*, two cornerstone bacteria in the oral microbiota associated with periodontal and peri-implant diseases. This represents a more ecologically valid pathogenic challenge than single-species or non-specific infections. Second, by pre-contaminating the titanium implant with this biofilm prior to surgical placement, it mimicked the critical clinical scenario of early bacterial colonization on the implant surface, which was the initial event leading to peri-implantitis. Therefore, the preventive efficacy demonstrated in this model enhanced the translational value of these findings.

When positioned within the current clinical landscape of peri-implantitis management, which often relies on mechanical debridement and local or systemic antibiotics with limited long-term success, the potential of Ag-Est816 becomes evident. Our previous *in vitro* study demonstrated that Ag-Est816 significantly reduced the *P. gingivalis*-*S. sanguinis* dual-species biofilms with no cell cytotoxicity (23). This nanocomposite offers a multimodal and targeted strategy that combines quorum quenching with localized bactericidal action. The mechanism of quorum-quenching enzyme in dispersing biofilm potentially creates a window of opportunity for the silver nanoparticles to penetrate more effectively and exert their direct bactericidal effects, which include membrane disruption, protein denaturation, and DNA damage (24–26). This approach, as supported by similar design principles in recent literature, is key to improving biocompatibility and achieving sustained, localized activity (19). Thus, Ag-Est816 could lead to more sustained disease control and reduce the recurrence rates commonly associated with conventional therapies. Based on the present *in vivo* findings, we hypothesize that the preventive effects may be consistent with the dual mechanism. This mechanistic interpretation remains to be directly validated within the complex *in vivo* environment. Future studies employing more direct analytical techniques on explanted biofilms (e.g., spatial mapping of QS signals, silver distribution, and biofilm architecture) are warranted to visually confirm this dynamic synergy *in situ*. Additionally, this study did not directly quantify the bacterial or biofilm burden on the explanted implants *in vivo*. Future studies would be strengthened by incorporating direct measures, such as sonication followed by CFU counts, quantitative PCR for bacterial DNA, or advanced imaging modalities (e.g., fluorescence *in situ* hybridization) to visually confirm biofilm reduction on retrieved implants.

The most significant outcome is the preservation of peri-implant bone. The marked improvement in BV/TV and Tb. N, coupled with histological evidence of new bone formation with intact trabeculae, indicates that Ag-Est816 effectively prevented infection-driven osteolysis and supported a bone reparative response (8, 27–29). The histological evidence further solidified this conclusion. The intact collagen matrix and normal bone morphology observed in Ag-Est816 group were hallmarks of a healthy bone remodeling process, in stark contrast to the rampant osteolysis and tissue degradation seen in infected group. While the precise temporal sequence and molecular interactions within the complex *in vivo* environment remain to be fully delineated, the observed functional outcome-effective bone preservation aligns with the intended preventive strategy of combining bactericidal and quorum quenching actions. In addition to the local biocompatibility evidenced by healthy bone formation, the histopathological assessment of major organs (heart, liver, spleen, lungs, and kidneys) from Ag-Est816 group showed no discernible pathological alterations compared to non-infected group. This provides preliminary evidence that the applied dose of Ag-Est816 did not elicit acute systemic toxicity over the 4-week study duration. However, these findings should be interpreted with caution, as qualitative histopathological assessment alone is insufficient to establish comprehensive safety. A more rigorous preclinical safety profile is required for translational development. Future studies must include extended-duration *in vivo* exposure with detailed hematological, biochemical, and quantitative histomorphometry analyses of organs. Furthermore, investigation into the long-term biodistribution and clearance kinetics of silver ions will be crucial. The favorable preventive outcome coupled with these initial biocompatibility findings strongly supports the justification for such in-depth safety investigations.

Despite these promising results, limitations of this study should be acknowledged. The primary limitation, as noted, is the anatomical and physiological difference between the rat femur and the human jawbone. The oral cavity presents a unique environment with a distinct resident microbiome, salivary components, and cyclic masticatory loads, all of which influence biofilm ecology, host immune response, and bone remodeling dynamics around dental implants. Our femoral model, by design, did not incorporate these oral-specific factors. Its purpose was to isolate and prove the core preventive principle-targeted biofilm disruption at the bone-implant interface-under standardized conditions. Therefore, the findings from this model represent a crucial proof-of-concept. Second, as noted, the dual-species biofilm is a purposeful simplification of the complex polymicrobial ecology in human peri-implantitis. This approach was essential to establish a clear cause-and-effect relationship between a defined, etiologically relevant pathogenic challenge (mimicking the critical early colonizer-keystone pathogen partnership) and the intervention. It allows for a precise mechanistic interpretation of Ag-Est816's effect. However, it does not fully capture the potential interspecies interactions, metabolic cross-feeding, or signaling redundancy that may occur in a diverse microbial community. Importantly, the Ag-Est816 strategy, combining broad-spectrum quorum quenching of AHLs with bactericidal silver action, is theoretically poised to address aspects of this complexity. Nonetheless, future studies employing polymicrobial biofilms derived from clinical samples and testing in intra-oral large animal models are imperative to validate the translational potential of this nanocomposite.

Furthermore, the 4-week observation period, while adequate to demonstrate the preventive potential of Ag-Est816 against the initial phase of infection-driven bone loss, does not address long-term outcomes critical for clinical application. The durability of the protective effect beyond the acute phase, the potential for late-onset local or systemic toxicity with sustained silver release, and the long-term risk of microbial adaptation or resistance remain open questions. We employed a conservative statistical approach regarding the independence of bilateral samples by using the animal as the primary experimental unit. The sample size, though yielding statistically significant results due to large effect sizes, is relatively small and may not fully capture broader biological variability. This limits the generalizability of the point estimates and underscores the need for confirmation in a larger, adequately powered study. Finally, the study does not include quantitative data on local inflammatory mediators. The observed reduction in bone loss is consistent with a dampened host inflammatory response to the pathogenic biofilm, a correlation supported by the histological absence of severe inflammatory infiltrate in the treatment group. However, direct measurement of key cytokines (e.g., TNF-α, IL-1β) or osteoclast activity markers (e.g., RANKL) would be required to mechanistically confirm this link. Therefore, future investigations will prioritize the analysis of the peri-implant inflammatory microenvironment to fully elucidate the immunomodulatory effects of Ag-Est816 and solidify its role in interrupting the critical “biofilm-inflammation-bone loss” axis in peri-implantitis. Addressing these points will be vital for advancing Ag-Est816 towards potential clinical use.

In conclusion, this study successfully established a modified rat femoral model to simulate early biofilm colonization on titanium implants. It provided proof-of-concept support for the potential of Ag-Est816 as an approach in preventing peri-implant bone loss and preserving structural integrity. This model does not replicate the full biological and clinical complexity of the human oral environment and therefore further validation in intra-oral animal models is essential to assess its translational relevance for clinical application.

## Data Availability

The original contributions presented in the study are included in the article/Supplementary Material, further inquiries can be directed to the corresponding author.
